# Exciton Dynamics in Layered Halide Perovskite Light‐Emitting Diodes

**DOI:** 10.1002/adma.202411998

**Published:** 2024-11-20

**Authors:** Sung‐Doo Baek, Seok Joo Yang, Hanjun Yang, Wenhao Shao, Yu‐Ting Yang, Letian Dou

**Affiliations:** ^1^ Davidson School of Chemical Engineering Purdue University West Lafayette IN 47907 USA; ^2^ Department of Chemical Engineering Kumoh National Institute of Technology 61 Daehak‐ro Gumi 39177 Republic of Korea; ^3^ Department of Chemistry Purdue University West Lafayette IN 47907 USA

**Keywords:** dynamics, excitons, halide perovskites, layered structure, light‐emitting diodes

## Abstract

Layered halide perovskites have garnered significant interest due to their exceptional optoelectronic properties and great promises in light‐emitting applications. Achieving high‐performance perovskite light‐emitting diodes (PeLEDs) requires a deep understanding of exciton dynamics in these materials. This review begins with a fundamental overview of the structural and photophysical properties of layered halide perovskites, then delves into the importance of dimensionality control and cascade energy transfer in quasi‐2D PeLEDs. In the second half of the review, more complex exciton dynamics, such as multiexciton processes and triplet exciton dynamics, from the perspective of LEDs are explored. Through this comprehensive review, an in‐depth understanding of the critical aspects of exciton dynamics in layered halide perovskites and their impacts on future research and technological advancements for layered halide PeLEDs is provided.

## Graphical abstract

This review discusses the exciton dynamics in layered halide perovskitelight‐emitting diodes (PeLEDs). It opens with a summary of the structural and photophysical aspects, followed by an exploration of controlling dimensionality and cascade exciton transfer in quasi‐2D PeLEDs. The review also addresses complex dynamics, such as multiexciton processes and triplet exciton dynamics, which are often overlooked.

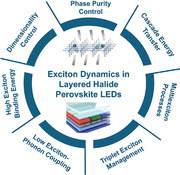

## 1 Introduction

Layered halide perovskites, particularly 2D and quasi‐2D structures, have garnered significant attention in recent years due to their exceptional optoelectronic properties and potential applications in light‐emitting devices, photovoltaics, and other technologies.^[^ ^1^, ^2^, ^3^, ^4^, ^5^, ^6^, ^7^ ^]^ These materials are characterized by their unique structural properties containing alternating lattices of organic and inorganic components, which significantly influence their photophysical behavior.^[^ ^8^, ^9^, ^10^ ^]^ The study of exciton dynamics in these materials is crucial for understanding and optimizing their performance in light‐emitting applications.

This review starts with a summary of the structural determinants and tunability of layered halide perovskites and then explores the fundamental photophysical considerations, focusing on the nature of excitons in layered halide perovskites, the effects of dimensional reduction on exciton binding energy, and the role of exciton–phonon coupling. Understanding these structural and photophysical determinants is key to manipulating excitonic behaviors and enhancing the material's functionality.

**Figure 1:**
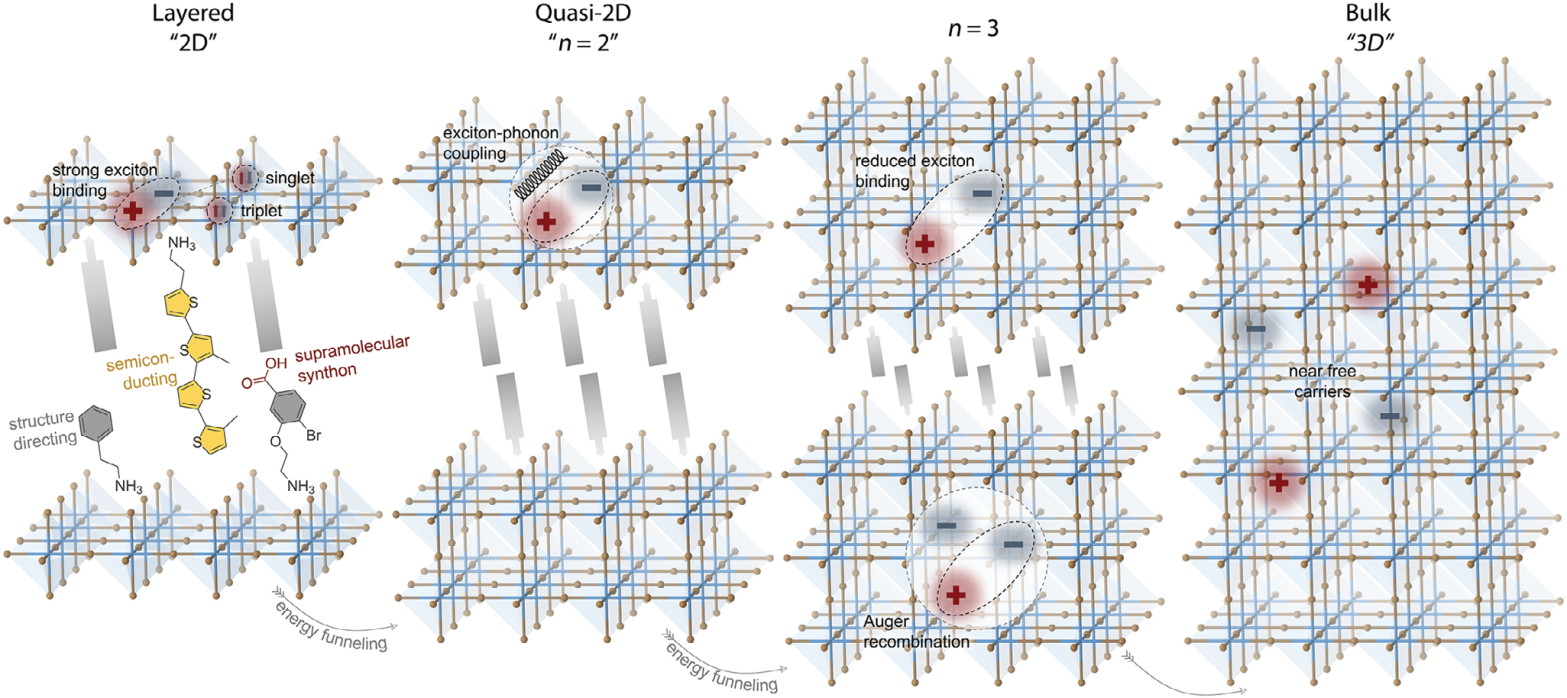
A schematic representation of the layered halide perovskites with the exciton dynamics discussed in this review.

This review further discusses the importance of dimensionality control and cascade energy transfer in quasi‐2D perovskite light‐emitting diodes (PeLEDs). Based on factors affecting photoluminescence quantum yield (PLQY), we also elaborate on strategies to control phase purity and approaches to optimize energy funneling. Additionally, multiexciton processes and triplet exciton dynamics will be explored, which are crucial for understanding the efficiency and stability of excitonic behaviors but have been slightly overlooked. Through this comprehensive overview, we aim to provide insights into the critical aspects of exciton dynamics in layered halide perovskites and their implications for future research and technological development of layered halide PeLEDs. A schematic representation of the layered halide perovskites with the exciton dynamics discussed in this review is shown in **Figure** **1**.

## 2 Structure Determinants and Tunability of Layered Halide Perovskites

Defined by a general formula of ABX_3_, bulk metal‐halide perovskite can be seen as an ionic assembly of negatively charged corner‐sharing metal‐halide octahedra ([BX_6_]^4−^), where B represents metal cations such as Pb^2+^ or Sn^2+^, while *X* denotes halide anions (Cl^–^, Br^–^, or I^–^), and positively charged, size‐restricted A‐site “fillings” like organic methylammonium (MA^+^), formamidinium (FA^+^), or inorganic Cs^+^. The transition from bulk to layered phase resembles an act of delamination along one crystallographic axis, resulting in octahedral lattices extending in the other two axes and stabilized with additional larger organic cationic spacers, often referred to as LA. The delamination can happen along the <100>, <110>, or <111> direction of the ideal cubic lattice of the bulk phase, and the choices of LA can be mono‐ or di‐cations. The prominent case discussed in the majority context here concerns the combination of a <100>‐terminated inorganic lattice and monoammonium cations due to their relatively easier crystal growth and superior excitonic behaviors. In this structural configuration of LA_2_A*_n_*_–1_B*_n_* X_3_ *_n_*_+1_ or LA_2_(ABX_3_)*_n–_*_1_BX_4_, *n* inorganic layers are intercalated between structure‐directing LAs, creating a multiple quantum‐well (MQW) structures. Herein, we use the popular terminology of 2D, quasi‐2D, and 3D halide perovskites to denote their layered *n* = 1, layered *n ≥* 2, and 3D (*n* = ∞) phases, respectively.

Unlike in 3D perovskites, where bulky organics are restricted to accommodating grain boundaries, the layered phases allow for the incorporation of complex organic spacers in a defined lattice, which subsequently inspires the molecular engineering of intermolecular interactions^[^ ^11^, ^12^ ^]^ and semiconducting organics with tunable energy gaps.^[^ ^13^ ^]^ These sophisticated spacers redefine the dielectric environment^[^ ^14^ ^]^ and exciton dynamics^[^ ^15^ ^]^ of layered halide perovskites.

## 3 Photophysical Considerations of Layered Halide Perovskites

### 3.1 Excitons in 2D Perovskites

**Figure 2:**
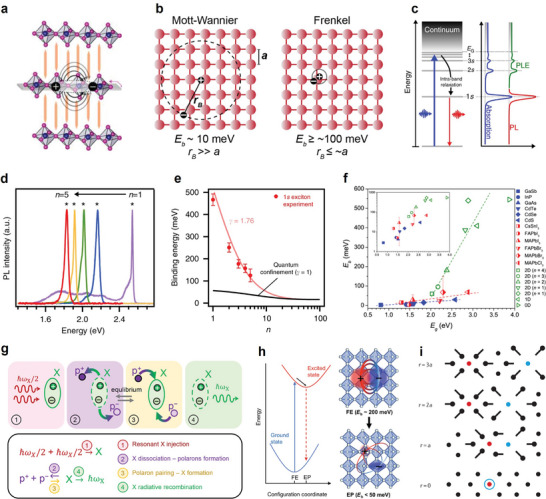
a) Structure of layered halide perovskites with natural quantum well systems. Reproduced with permission.^[^ ^16^ ^]^ Copyright 2019, Elsevier. b) Schematics of the excitons of the Mott–Wannier and Frenkel types in an arbitrary atomic lattice. Reproduced under terms of the CC‐BY license.^[^ ^18^ ^]^ Copyright 2016, Manser et al. American Chemical Society. c) Illustrations of the Rydberg series of the exciton ground state. d) Photoluminescence spectra observed in perovskites with varying well thicknesses. e) Scaling law of the exciton binding energy with the perovskite layer thickness. Reproduced under terms of the CC‐BY license.^[^ ^20^ ^]^ Copyright 2018, Blancon et al. Springer Nature. f) Empirical relationship between *E*_g_ and *E*_b_ in several 3D and quasi‐2D perovskites. Reproduced under terms of the CC‐BY license.^[^ ^18^ ^]^ Copyright 2016, Manser et al. American Chemical Society. g) Schematics of exciton dissociation into free carriers and equilibrium dynamics. Reproduced under terms of the CC‐BY license.^[^ ^24^ ^]^ Copyright 2023, Simbula et al. Springer Nature. h) Configuration space representation of potentials of the ground state and excited state with different equilibrium positions under the harmonic approximation. Reproduced under terms of the CC‐BY license.^[^ ^25^ ^]^ Copyright 2020, Tao et al. American Association for the Advancement of Science. i) A schematic showing the short‐range, repulsive overlap between electron and hole polarons. Reproduced under terms of the CC‐BY license.^[^ ^26^ ^]^ Copyright 2021, Franchini et al. Springer Nature.

Exciton refers to the bound state of excited electrons and holes, which are attracted to each other by Coulombic forces. The minimum energy required to ionize this bound electron–hole pair from the lowest energy eigenstate into uncorrelated free charge carriers is called the exciton binding energy.^[^ ^16^ ^]^ Dimensionality reduction from bulk to layered halide perovskites is accompanied by reduced dielectric screening and boosted quantum confinement (**Figure** **2**a),^[^ ^16^ ^]^ which enhances the strength of Coulombic interaction and ultimately leads to strongly bound electron–hole pairs.^[^ ^17^ ^]^

Descriptions of excitons in halide perovskites usually adopt the classic Frenkel and Wannier models (Figure 2b).^[^ ^18^ ^]^ Typically, Wannier excitons possess binding energy of 10–30 meV, whereas Frenkel excitons are of 500–1000 meV.^[^ ^19^ ^]^ When their binding energy is comparable to the thermal fluctuation energy at room temperature (≈26 meV), excitons tend to dissociate into free carriers with longer diffusion lengths. Experimentally (Figure 2c), the exciton binding energy can be simply extracted from the exciton Rydberg series by measuring optical absorption, PL, and PL excitation spectroscopy.^[^ ^20^ ^]^ Under this model, excitons in 3D perovskites usually adopt Wannier characters with binding energies of 2–60 meV.^[^ ^21^ ^]^ In comparison, 2D and quasi‐2D perovskites possess the so‐called “Frenkel–Wannier hybrid excitons”. The MQW structure and the relatively insulating organic spacers give rise to strongly confined Wannier excitons in the inorganic framework and the Frenkel excitons are more likely generated in the surrounding bulky organic spacers.^[^ ^19^, ^22^ ^]^

Therefore, strongly bound excitons with binding energy of up to a few hundreds of meV are the major excited‐state species in low‐dimensional perovskites. However, with the inclusion of semiconducting organic cations,^[^ ^23^ ^]^ strong electron‐accepting candidates are thus able to increase the electrostatic screening of excitons and lower their binding energy.^[^ ^14^ ^]^ On the other hand, the energy gap of the organic lattice can be engineered^[^ ^15^ ^]^ with respect to the inorganic and thus confine the exciton in the organic lattice or spontaneously separate the electron–hole pair at the interface. These candidates showcase the unique tunability of an organic–inorganic hybrid structure and find their value in light‐emitting applications.

### 3.2 Exciton Energy from 2D to Quasi‐2D Perovskites

Originating from the different exciton dynamics between 3D and 2D perovskites, the photophysical properties of quasi‐2D phases are tunable by controlling the inorganic well thickness (*n*‐value). This enables facile emission color tunability (Figure 2d), which is one of the most important advantages of layered halide perovskites.^[^ ^20^ ^]^ As depicted in Figure 2e, Mohite and colleagues developed a scaling rule for exciton binding energy as a function of *n*, which accounts for dielectric confinement effects.^[^ ^20^ ^]^ On the other hand, Manser and colleagues summarized the empirical relationship between the band gap and exciton binding energy in several 3D and quasi‐2D perovskites (Figure 2f).^[^ ^18^ ^]^ By increasing the *n*‐values, both observed a rapid decline in the exciton binding energies in the quasi‐2D perovskites, demonstrated in a series of quasi‐2D perovskite compounds featuring different bulky organic spacers.

### 3.3 Polaron and Exciton–Phonon Interaction

However, owing to the soft and polarizable halide perovskite lattices, electrons or holes can be easily coupled with lattice vibration to form polaron pairs. This exciton‐lattice phonon interaction lowers the carrier mobility, increases its effective mass, and plays a crucial role in exciton dynamics. As depicted in Figure 2g, Simbula and colleagues summarized a phonon‐incorporated exciton dynamic picture.^[^ ^24^ ^]^ After vertical excitation, excitons form and relax to an excited‐state equilibrium configuration between the bound and the exciton polaron state. In the latter, excitons are dissociated and screened by the atomic displacement of nearby lattice to form polaron pairs (p^+^ or p^–^). Consequently, the polaronic effect lowers the exciton binding energy. Light emission occurs after equilibrium is reached, as the excitons reassociate and undergo radiative recombination.

Furthermore, polaronic effects can be classified as long‐range electrostatic polarization response (Fröhlich‐like interaction) and short‐range deformation potential (Holstein‐like interaction).^[^ ^26^ ^]^ Typically, 3D perovskites show a long‐range Fröhlich‐like exciton–phonon interaction, where free charge carriers are thought to exist as large polarons.^[^ ^27^ ^]^ These large polarons account for the low electron–hole recombination rate and high defect tolerance in 3D perovskites. In comparison, layered halide perovskites possess an enhanced short‐range Holstein‐like interaction due to the reduced dimensionality and increased lattice distortion.^[^ ^28^ ^]^ Consequently, excitons in layered halide perovskites exhibit significant polaronic characteristics and exist as exciton polarons.^[^ ^29^ ^]^ This model is intuitively illustrated in Figure 2h by Tao et al.^[^ ^25^ ^]^ Subsequently, the opposite lattice deformations of the electron polaron and hole polaron are observed, which separate the electron and hole wave functions in real space (Figure 2i).^[^ ^26^ ^]^ Therefore, the electron–hole wave function overlaps, and the exciton binding energy is dramatically reduced, which weakens intra‐ and inter‐exciton interactions. Briefly, the polaronic effects strongly renormalize excited‐state carrier behaviors due to lattice anharmonicity and dynamic disorder in both 3D and 2D perovskites.

### 3.4 Self‐Trapped Exciton (STE)

**Figure 3:**
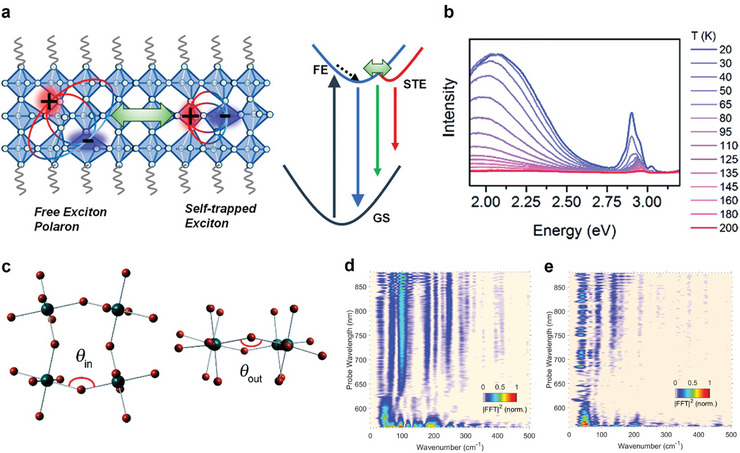
a) Schematics of self‐trapped electrons originating from exciton polaron. Reproduced with permission.^[^ ^29^ ^]^ Copyright 2022, American Chemical Society. b) Broad emission due to lattice reorganization and free exciton emission observed in 2D perovskites. c) In‐plane and out‐of‐plane octahedral distortion which leads to broad emission. Reproduced with permission.^[^ ^30^ ^]^ Copyright 2017, American Chemical Society. Wavelength‐resolved Fourier transform power map of d) butylammonium lead iodide and e) hexylammonium lead iodide. Reproduced under terms of the CC‐BY license.^[^ ^31^ ^]^ Copyright 2017, Ni et al. American Chemical Society.

Notably, the exciton–phonon interaction can cause local lattice distortion, trapping excitons in a low‐energy self‐trapped exciton (STE) state.^[^ ^32^ ^]^ When a charge carrier distorts the surrounding structural lattice, a local potential well is formed, and the charge carrier becomes self‐trapped.^[^ ^33^ ^]^ While free exciton polarons (with respect to the trap state) are usually stable in 3D perovskites, due to the strong short‐range exciton–phonon interactions in 2D perovskites, self‐trapped states are more likely to be in thermal equilibrium with free states (**Figure** **3**a).^[^ ^29^ ^]^ This means that both states coexist, and dynamic exchange between them is possible. Therefore, exciton polarons in 2D perovskites experience ultrafast dynamic interconversion between free and self‐trapped states. Consequently, broad emission with large Stokes shifts is observed as an intrinsic phenomenon in 2D perovskites (Figure 3b).^[^ ^30^ ^]^

From a structure perspective, Karunadasa's group demonstrated that the broad emission in perovskites was strongly correlated with the in‐plane and out‐of‐plane inorganic lattice distortion (Figure 3c).^[^ ^30^ ^]^ The strength of exciton–phonon interaction can also be affected by the choice of organic cations, which determines the vibrational modes the exciton couples to (Figure 3d,e).^[^ ^31^ ^]^ For instance, in butylammonium lead iodide, excitons predominantly couple to a 100 cm^−1^ phonon mode, whereas in hexylammonium lead iodide, excitons interact with phonons with frequencies of 88 and 137 cm^−1^.

## 4 Dimensionality Control and Cascade Energy Transfer in Quasi‐2D PeLEDs

Quasi‐2D halide perovskites, which combine the high exciton binding energy characteristic of 2D perovskites with the reduced exciton–phonon interactions typical of 3D structures, have attracted widespread interest in PeLEDs. The former enhances radiative recombination, while the latter suppresses non‐radiative loss channels, collectively leading to higher PLQY.

### 4.1 Factors Determining PLQY

The PLQY of halide perovskites can be approximated as:

(1) $$\mathrm{PLQY}\left(N\right)=\frac{k_{1,\mathrm{exci}\mathrm{ton}}+k_{2,\mathrm{rad}}N}{k_{1,\mathrm{exci}\mathrm{ton}}+k_{2,\mathrm{rad}}N+k_{1,\mathrm{trap}}+k_{2,nr}N+k_{3}N^{2}}$$

where *k_1,exciton_* is the monomolecular radiative recombination constant of excitons, *k_2,rad_* is the bimolecular radiative recombination constant of free carriers, *k_1,trap_* is the trap‐assisted nonradiative monomolecular recombination constant, *k_2,nr_* is the bimolecular nonradiative recombination constant, *k_3_* is the Auger recombination constant, and N is the carrier concentration. In quasi‐2D perovskites, *k_2,nr_* is relatively low due to the dominance of trap‐assisted and Auger recombination as the primary non‐radiative pathways.^[^ ^34^, ^35^ ^]^

**Figure 4:**
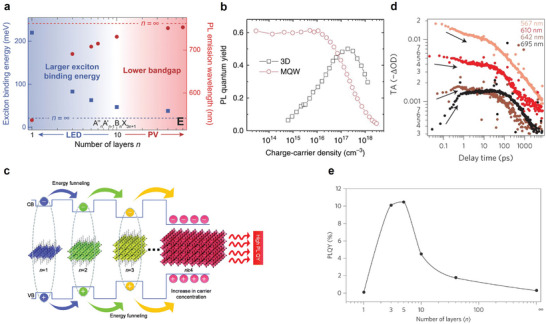
a) Exciton binding energy and PL emission wavelength depending on *n*‐values. Reproduced with permission.^[^ ^38^ ^]^ Copyright 2018, Wiley‐VCH b) Carrier density‐dependent PLQYs of films measured with 405 nm laser pulses. Reproduced under terms of the CC‐BY license.^[^ ^35^ ^]^ Copyright 2017, Xing et al. Springer Nature. c) Illustration of efficient energy transfer from large bandgaps to low bandgaps. Note that CB and VB are conduction and valence bands, respectively. Reproduced with permission.^[^ ^49^ ^]^ Copyright 2021, Royal Society of Chemistry. d) Ground‐state bleaching (GSB) peaks in TA spectra as a function of delay time, representing the energy transfer. e) PLQY trend from different *n*‐values. Reproduced under terms of the CC‐BY license.^[^ ^50^ ^]^ Copyright 2016, Yuan et al. Springer Nature.

Therefore, *k_1,trap_* and *k_3_N^2^* together contribute to the nonradiative processes, while *k_1,exciton_* and *k_2,rad_N* jointly constitute the radiative recombination in quasi‐2D perovskites, which include both excitonic and free carrier contents due to their multidimensionality. This characteristic distinguishes quasi‐2D perovskites from their 2D and 3D counterparts when the carrier concentration is tuned by varying the current density or photoexcitation power. One would therefore expect the excited state kinetics to be tuned with *n* phase control. The high exciton binding energy in the *n *= 1 phase (**Figure** **4**a) makes *k_1_* ​ approximately three times larger than that in *n *= 2–4.^[^ ^36^, ^37^, ^38^ ^]^ However, because of stronger electron–phonon interactions in *n* = 1 phase, the PLQY of 2D perovskite is lower than that of quasi‐2D perovskites with intermediate *n*‐values.^[^ ^39^, ^40^, ^41^ ^]^ A typical power‐dependent PLQY curve of low‐*n* dominant quasi‐2D perovskite is shown in Figure 4b, which exhibited a plateau at *N* below 10^16^ cm^−3^, as *N*‐independent *k_1,exciton_* dominates over other processes.^[^ ^35^ ^]^ In contrast, the PLQY of 3D perovskite increased with rising carrier density since carrier recombination was dominated by free carriers (i.e., *k_2,rad_N* dominating). At high carrier densities, Auger recombination *k_3_N^2^* becomes dominant in both cases, leading to nonradiative recombination. This process will be discussed in detail in Section 5. Additionally, *k_1,trap_* is a critical parameter influencing the PLQY in quasi‐2D perovskites. In this regard, trap‐assisted recombination can be effectively reduced by controlling the *n*‐phase distribution, a key focus of Section 4, which contrasts with the direct defect passivation commonly employed in 3D perovskites. In quasi‐2D perovskites, charge carriers preferentially undergo energy transfer between uniform phase distribution rather than being trapped at defect sites. Consequently, efficient energy funneling not only facilitates energy transfer but also significantly reduces trap‐assisted recombination, thereby enhancing the PLQY in quasi‐2D perovskites.^[^ ^42^, ^43^, ^44^, ^45^, ^46^, ^47^, ^48^ ^]^

### 4.2 Importance of Phase Purity and Distribution Engineering

One would then imagine that delicate dimensionality control is critical to achieving high PLQY and tunable emission color in quasi‐2D PeLEDs. However, attaining phase purity remains a significant challenge for polycrystalline perovskite films. On the thermodynamics front, quasi‐2D perovskites tend to undergo phase separation into 2D and 3D counterparts due to the low formation energy of 2D phases.^[^ ^51^, ^52^, ^53^ ^]^ This challenge, commonly known as 2D/3D phase separation, must be mitigated as it can result in non‐uniform emission.^[^ ^54^ ^]^ Additionally, various intermediate complexes in the precursor solution nucleate upon rapid film crystallization depending on the precursor solution stoichiometry, solvent, and fabrication method in quasi‐2D perovskites.^[^ ^55^ ^]^ These intermediate species subsequently grow into a broad distribution of *n* phases in the final film. To suppress the phase separation, several studies have reported crystallization techniques, such as hot‐casting methods or slow crystallization methods in other device applications.^[^ ^56^, ^57^, ^58^, ^59^, ^60^, ^61^, ^62^, ^63^ ^]^

Despite phase purity issues, PLQY of quasi‐2D perovskites can still be strategically enhanced through efficient energy funneling within a continuous *n*‐phase distribution (Figure 4c).^[^ ^49^, ^64^, ^65^, ^66^, ^67^, ^68^, ^69^, ^70^, ^71^, ^72^, ^73^, ^74^, ^75^ ^]^ In quasi‐2D perovskite structures where different *n*‐values coexist, charge carriers tend to migrate from higher‐energy (low‐*n*) layers to lower‐energy (high‐*n*) layers. This migration results in charge accumulation in the lower‐energy regions, where radiative recombination and light emission are predominant. The energy concentration resulting from this cascade effect reduces non‐radiative recombination, thereby significantly enhancing PLQYs. Due to the energy cascade from low to high *n* phases, an efficient energy transfer reduces non‐radiative recombination and concentrates carrier population at the lowest bandgap phase. This process can be experimentally tracked with transient absorption (TA) spectra. As shown by Yuan et al., four distinct bleaching peaks emerged over time in a quasi‐2D perovskite film synthesized with an initial stoichiometry of *n *= 3 (Figure 4d).^[^ ^50^ ^]^ The decreased TA signals at 567 and 610 nm and the emergence of 642 and 695 nm signals corresponded to the cascade energy transfer from low to high *n* phases. Interestingly, the highest PLQY was observed at *n*‐values between 3 and 5 instead of *n *= 1 (2D), *n *> 10, or 3D perovskites (Figure 4e).^[^ ^50^ ^]^ The relatively high PLQYs at these intermediate *n*‐values are again attributed to the different energy landscapes.^[^ ^76^ ^]^ In a flat energy landscape, as seen in 3D perovskites, carriers diffuse and recombination predominantly occurs at grain boundaries, reducing radiative recombination. In contrast, efficient energy funneling in an *n*‐optimized landscape bypasses low *n* phases with high exciton–phonon coupling and thus enhances radiative recombination efficiency.

### 4.3 Strategies to Suppress 2D/3D Phase Separation

**Figure 5:**
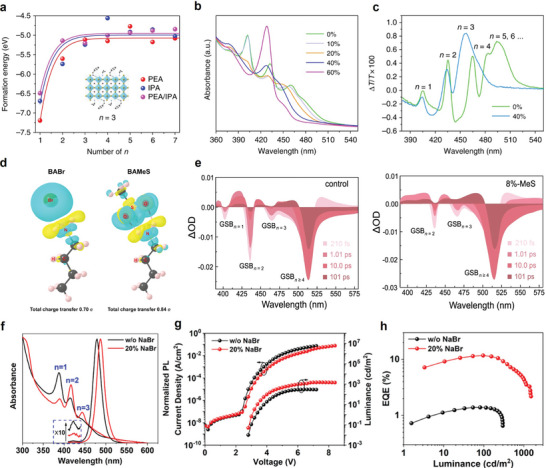
a) Formation energy of quasi‐2D perovskites composed of PEA, IPA, and PEA/IPA with different *n*‐values. b) Absorption spectra of the PEA_2_A_1.5_Pb_2.5_Br_8.5_ film with different ratios of IPABr. c) TA spectra of the PEA_2_A_1.5_Pb_2.5_Br_8.5_ film with 0% and 40% of IPABr. Reproduced under terms of the CC‐BY license.^[^ ^52^ ^]^ Copyright 2018, Xing et al. Springer Nature. d) A schematic of the interaction between BA and Br and between BA and MeS. e) TA spectra of the control and 8%‐MeS treated films at different delay times. Reproduced under terms of the CC‐BY license.^[^ ^77^ ^]^ Copyright 2021, Kong et al. Springer Nature. f) Absorption spectra of quasi‐2D perovskite films without and with 20% NaBr. g) Current density (*J*)–voltage (*V*)–luminance (*L*) curve and h) EQE–luminance characteristics of quasi‐2D PeLEDs without and with 20% NaBr. Reproduced with permission.^[^ ^78^ ^]^ Copyright 2020, American Chemical Society.

The suppression of *n *= 1 phase (2D perovskite) formation is important to achieve high PLQY. In this regard, Xing et al. doped isopropylammonium (IPA) alongside phenylethylammonium (PEA) to restrict 2D phase formation due to the steric hindrance restrictions of the primary ammonium in IPA.^[^ ^52^ ^]^ Interdigitating IPA doping increased the calculated 2D perovskite formation energy to −6.5 eV compared to when only PEA (−7.2 eV) or IPA (−6.7 eV) are employed (**Figure** **5**a). This trend was also confirmed by film absorption curves showing the suppression of the 2D phase upon IPA substitution (Figure 5b). TA spectra revealed the suppression of high *n* and 3D phases as the *n *≥ 4 phase signals disappeared in the sample with 40% IPA substitution (Figure 5c).

Kong et al. controlled the crystallization by doping methanesulfonate (MeS) as a form of CsMeS.^[^ ^77^ ^]^ MeS binds to the organic ammonium site (butylammonium, BA in this case) more strongly than BA^+^–Br^−^ binding through multiple hydrogen bonds (Figure 5d), thus reducing the formation of the 2D perovskites based on BA. TA analysis of perovskite film with 8% MeS doping also supported the elimination of the ground‐state bleaching (GSB) signal from *n *= 1 phase (Figure 5e). Additionally, after 101 ps, the control retained excitons from *n *= 2 phase while only *n *≥ 4 phases remained in the MeS‐doped sample, indicating a more efficient cascade energy transfer. As a result, a high‐efficiency green LED with an external quantum efficiency (EQE) exceeding 20% was reported.

Furthermore, Pang et al. optimized the film formation of a quasi‐2D perovskite by incorporating 20% sodium bromide (NaBr) into the precursor solution.^[^ ^78^ ^]^ PEA and Na ions competed with each other during the crystallization, preventing the formation of PEA‐incorporated 2D perovskites. Absorption spectra revealed the decreased 2D signal and increased *n *= 2 and 3 phases upon NaBr addition (Figure 5f). They explained that the resulting red‐shifted PL may be attributed to the recovery of 3D nature or reduced Cl content of the emitting domains. Luminance increased significantly in the NaBr‐treated quasi‐2D PeLEDs, with EQE rising from 1.4% to 11.7% (Figure 5g,h).

### 4.4 Strategies to Suppress Ion Migration for Phase Distribution Engineering

**Figure 6:**
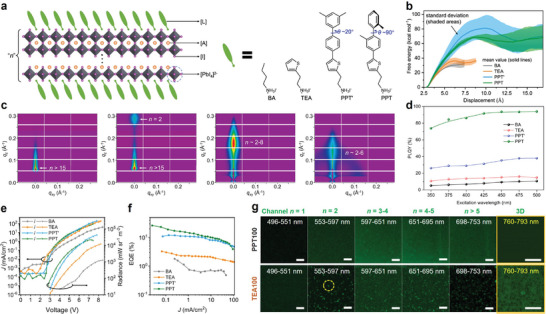
a) A schematic of large organic cations of BA, TEA, PPT’, and PPT in the quasi‐2D perovskite structure. b) Free energy profile of the direct diffusion for I^−^ into large organic layers. c) GISAXS pattern of the BA, TEA, PPT’, and PPT‐based quasi‐2D perovskite films with *n‐* values. d) PLQYs versus excitation wavelengths of the BA, TEA, PPT’, and PPT‐based quasi‐2D perovskite films. e) *J*–V–radiance (*R*) characteristics and f) EQE versus *J* curve of the BA, TEA, PPT’, and PPT‐based quasi‐2D PeLEDs. Reproduced under terms of the CC‐BY license.^[^ ^80^ ^]^ Copyright 2023, Wang et al. Springer Nature. g) Confocal PL mapping of PPT and TEA‐based quasi‐2D perovskite films with different wavelength ranges. Reproduced with permission.^[^ ^81^ ^]^ Copyright 2023, American Chemical Society.

The second effective strategy to engineer phase distribution in quasi‐2D perovskites is to decrease ion migration via the molecular design of organic spacers. Thermal annealing during crystallization causes all compositions to diffuse, transforming to higher *n* phases regardless of the initial stoichiometry.^[^ ^79^, ^80^, ^81^ ^]^ Therefore, phase separation in quasi‐2D perovskites can be effectively controlled by suppressing ion migration. Wang et al. comprehensively studied four organic spacers in a quasi‐2D perovskite with *n *= 3 initial stoichiometry: BA, thiophenylethylammonium (TEA), 2‐(5‐(3′,5′‐dimethyl‐[1,1′‐biphenyl]−4‐yl)thiophen‐2‐yl)ethyl‐1‐ammonium (PPT’), and 2‐(5‐(2,2′‐dimethyl‐[1,1′‐biphenyl]−4‐yl)thiophen‐2‐yl)ethyl‐1‐ammonium (PPT) (**Figure** **6**a).^[^ ^80^ ^]^ The design of PPT’ and PPT, both containing two phenyl groups and one thiophene, focused on increasing the hydrophobicity of the molecular backbone while “closing” the intermolecular spaces to prevent mass transfer (ion migration) during perovskite crystal growth that gave rise to phase separation. The molecular backbone lengths and cross‐sectional areas both increased substantially from BA through TEA to PPT’ and PPT. Notably, PPT presented a more twisted molecular backbone compared with PPT’ due to the additional steric hindrance between two methylated phenyl units, leading to its even larger molecular cross‐sectional area.

To assess the impact on ion migration, the free energy of direct diffusion into the bulky organic layers for iodide ion (I^–^) was calculated using molecular dynamics (MD) simulation (Figure 6b). The free energy required for I^−^ diffusion into the organic layers is approximately twice as high for PPT and PPT' layers compared to BA and TEA layers. Although this estimation is simplified at *n *= 1, the differences in ion migration within quasi‐2D perovskites are also expected to be significant. The difference in *n* distribution resulting from the suppressed ion migration was clearly revealed by grazing incidence small‐angle X‐ray scattering (GISAXS) analysis (Figure 6c). BA and TEA‐based quasi‐2D perovskite films both contained high *n* phases (>15), while the PPT’‐based film exhibited an *n*‐distribution from 2 to 8, which was further narrowed down to 2–6 with PPT. This narrow phase distribution gave rise to a superior cascade energy funneling path in the PPT‐based film, resulting in a high PLQY of 94% compared to 40%, 15%, and 10% when PPT’, TEA, and BA were used, respectively (Figure 6d). In addition, quasi‐2D PeLEDs utilizing PPT exhibited the lowest turn‐on voltage of 2.8 V, showing additional defect passivation (Figure 6e). Consequently, a high EQE of 26.3% was achieved for quasi‐2D PeLEDs with red emission at 700 nm (Figure 6f).

Yang et al. subsequently visualized the phase distribution using confocal laser scanning microscopy, further supporting a narrow *n* distribution in the PPT‐based films from 3 to 5, whereas aggregated *n *= 2 and a dominant 3D phase coexisted in TEA‐based films (Figure 6g).^[^ ^81^ ^]^ This non‐uniform TEA film hinders energy transfer and reduces the color purity of emission. This study additionally explored another bulky organic spacer, 2‐(2′′,3′‐dimethyl‐[1,1′:4′,1′′‐terphenyl]‐4‐yl)ethyl‐1‐ammonium (PPP), featuring a similar backbone twisting as PPT but with three phenyl groups. The PPP‐based quasi‐2D film also exhibited a high PLQY close to 90% and an EQE exceeding 20% in the LED devices. Furthermore, results also demonstrated that PPT and PPP spacers could suppress ion migration in halide‐alloyed quasi‐2D perovskite films and achieve color‐stable PeLEDs with tunable emission wavelengths. However, the *n*‐phase separation was accelerated when Br content increased in the I‐dominant system. Halide mixing ratios must be carefully managed in quasi‐2D perovskites to prevent unwanted *n*‐phase separation. Overall, a comprehensive comparison of the recent two studies indicated this useful strategy to suppress ion migration, control phase distribution, and ensure efficient energy cascade via molecular backbone design of organic spacers to increase their bulkiness.^[^ ^80^, ^81^ ^]^

### 4.5 Other Strategies for Efficient Energy Funneling

**Figure 7:**
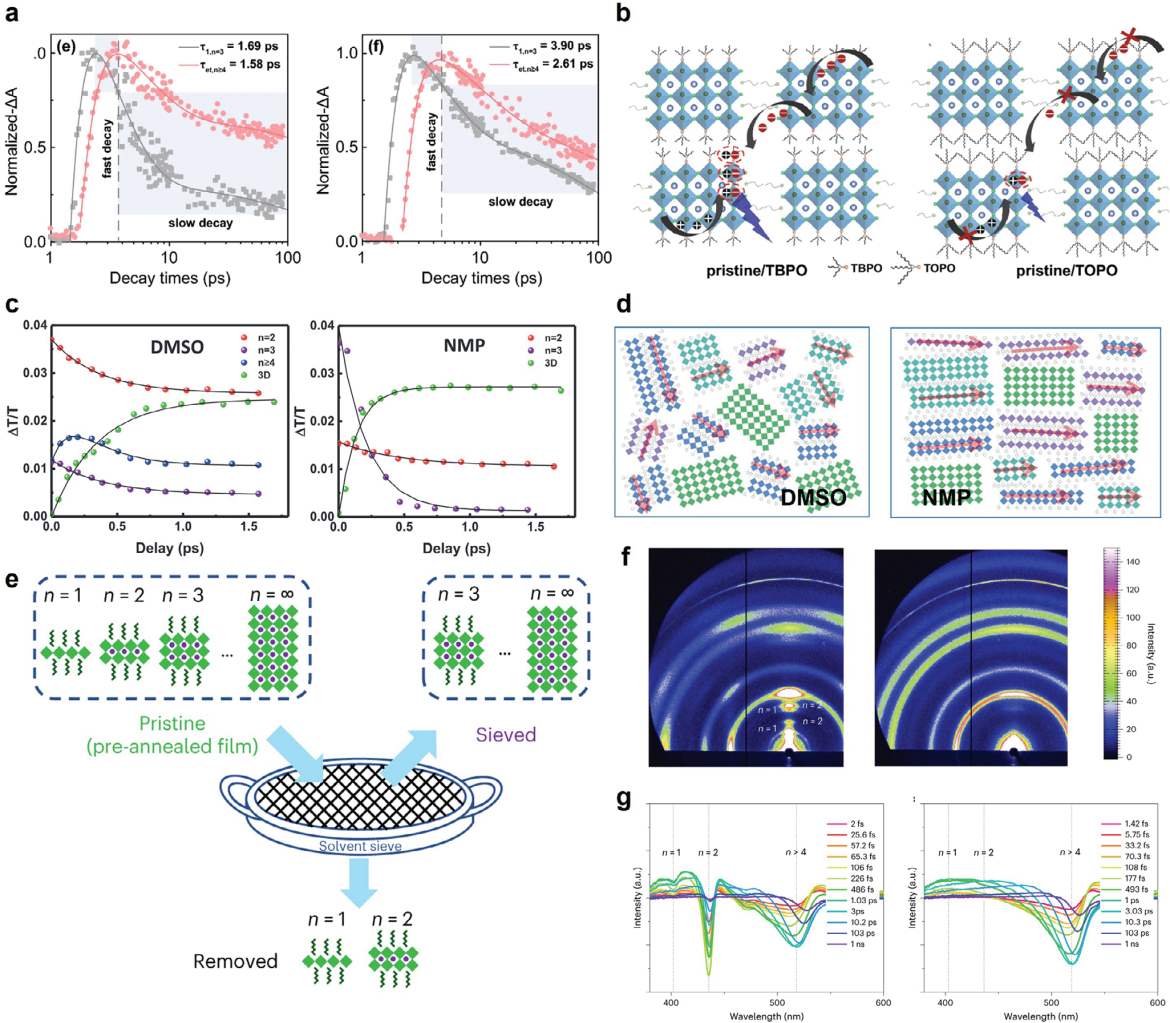
a) GSB peaks corresponding to *n* = 3 and ≥4 extracted from TA spectra as a function of decay times with TBPO and TOPO additives. b) Schematics of the different mechanisms of the energy transfer between the TBPO and TOPO‐treated films. The mediated carbon chain enhances the energy transfer between the crystal domains. Reproduced under terms of the CC‐BY license.^[^ ^82^ ^]^ Copyright 2022, Ren et al. Springer Nature. c) All GSB peaks in TA spectra are replotted as a function of delay times for the quasi‐2D perovskite films synthesized via DMSO and NMP. d) Schematics of the efficient energy transfer depending on the crystal orientation from different organic solvent types. Reproduced with permission.^[^ ^83^ ^]^ Copyright 2020, Wiley‐VCH. e) Illustration of the solvent sieve method. f) GIWAXS mapping and g) TA spectra of the pristine (left) and the solvent‐sieved film (right). Reproduced under terms of the CC‐BY license.^[^ ^85^ ^]^ Copyright 2024, Ding et al. Springer Nature.

Ren et al. compared a series of phosphine oxides with different carbon chain lengths – triethylphosphine oxide (TEPO), tributylphosphine oxide (TBPO), and trioctylphosphine oxide (TOPO) – as defect passivation agents in quasi‐2D perovskites to suppress trap‐assisted nonradiative recombination.^[^ ^82^ ^]^ Electron pairs in the P═O functional group significantly reduced non‐radiative recombination by passivating Pb^2+^. Consequently, the PLQY of the quasi‐2D perovskite was improved from 50.3% (pristine) to 56.4%, 63.8%, and 69.8% with TEPO, TBPO, and TOPO, respectively. TA spectra revealed three GSB peaks at 429, 453, and 480 nm, corresponding to *n *= 2, 3, and ≥4, respectively. Over a few ps, the GSB peak of *n *= 3 decreased while that of *n *≥ 4 increased. The fast decay (*τ_1_*) and formation time constant (*τ_et_*) were fitted using a multiexponential function. *τ_1_* at *n *= 3 was shortened from 3.90 ps in the TOPO film to 1.69 ps in the TBPO film, and *τ_et_* for *n *≥ 4 was also shortened from 2.6 ps in the TOPO film to 1.58 ps in the TBPO film (**Figure** **7**a). The fast energy transfer in the TBPO film indicated that a moderate chain length of the passivation agent at the grain boundary was crucial for energy funneling (Figure 7b), resulting in a high EQE of 11.5% for blue LED, which was the highest reported at that time.

The orientation of quasi‐2D perovskite crystals also significantly affects energy transfer. Lei et al. investigated the influence of organic solvents, dimethyl sulfoxide (DMSO), and N‐methyl‐2‐pyrrolidone (NMP) on crystal orientation.^[^ ^83^ ^]^ Both solvents acted as Lewis bases, which enhanced perovskite crystallinity by forming an intermediate phase with Pb^2+^.^[^ ^84^ ^]^ However, their different surface wettability, evaporation rates, and intermediate phase types influenced crystal dimension and orientation. In grazing incidence wide‐angle X‐ray scattering (GIWAXS) analysis, the DMSO‐treated perovskite film exhibited a random or slightly perpendicular orientation, predominantly with *n *= 2 phase. In contrast, the NMP‐treated film showed in‐plane alignment with coexisting *n *= 1 and 2 phases. TA spectra revealed various GSB peaks, including 3D phases as the ultimate emitting domain. Energy transfer dynamics were analyzed by plotting GSB signals against time (Figure 7c). For the DMSO‐treated film, *n *= 2 and 3 phases displayed decay time constants of ≈0.3 ps, while *n *≥ 4 phase showed a rise time constant of 0.2 ps followed by a slow decay constant of 0.4 ps, and the 3D phase exhibited a rise time of 0.36 ps. Meanwhile, the NMP‐treated film contained no visible *n *≥ 4 GSB peaks, and the 3D rise time constant was merely 0.13 ps, indicating much faster energy transfer compared to the DMSO‐treated film. This accelerated energy transfer in the NMP‐treated film resulted from its aligned crystal orientation, which affected the preferential transition dipole orientation and increased grain density due to reduced spacing (Figure 7d).

More recently, Ding et al. achieved efficient energy funneling by removing low *n* phases through a solvent sieve method.^[^ ^85^ ^]^ Low *n* phases were found to correlate with defect formation and therefore reduced the stability of quasi‐2D perovskite films.^[^ ^86^, ^87^ ^]^ The solvent sieve method involves adding 0–5% of polar hexylamine (HA) to the non‐polar primary solvent chlorobenzene (CB), and treating the pristine film with the mixed solvent, resulting in the elimination of low *n* phases (Figure 7e). GIWAXS exhibited the elimination of 2D signals and nearly diminishing *n *= 2 phases in the sieved film (Figure 7f), on which the TA analysis additionally indicated the disappearance of GSB peaks for low *n* phases and a reduction in the rise time for *n *≥ 4 phases to reach maximum intensity from over 3 ps (pristine) to 1 ps (Figure 7g). Via fast energy funneling with the reduction of low *n* phases that induce instability, green PeLEDs demonstrated a high EQE of 29.5% and an estimated stability of 5.7 years at 100 cd m^−^^2^.

Ongoing studies continued to explore and report various molecular additives to further enhance the efficiency of energy funneling in quasi‐2D PeLEDs. For instance, crown molecule (e.g., 1,4,7,10,13,16‐hexaoxacyclooctadecane) was shown to facilitate rapid energy funneling by reducing crystal size and ensuring uniform size distribution.^[^ ^88^ ^]^ ABA (4‐(2‐Aminoethyl)benzoic acid) enhanced energy transfer via robust interactions between quasi‐2D perovskite domains, mediated by carboxylic and amino groups.^[^ ^89^ ^]^ In addition, it has been reported that sodium 4‐fluorobenzoate (SFB) plays a critical role in inhibiting the formation of 2D phases, thereby promoting energy transfer through *n *= 2 and *n *= 3 phases.^[^ ^90^ ^]^ The incorporation of carbon dots into a bottom layer is another method to mitigate the formation of 2D phases during crystallization and reduce conductivity in the bottom layer, which is essential for charge balance.^[^ ^91^ ^]^

### 4.6 Phase‐Pure Quasi‐2D PeLEDs

**Figure 8:**
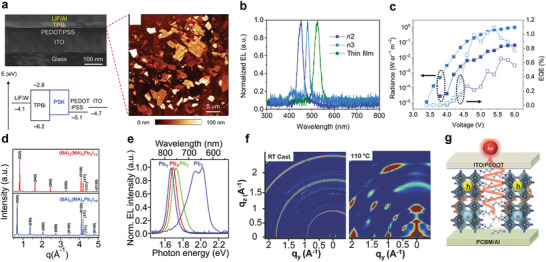
a) Cross‐sectional scanning electron microscope image of the single crystal quasi‐2D PeLED with atomic force microscopy image of the single crystal nanosheets and energy level of the interlayers of the device. b) EL spectra and c) radiance–voltage–EQE curves of the single crystal quasi‐2D PeLEDs of *n *= 2 and 3 with the pristine film. Reproduced under terms of the CC‐BY license.^[^ ^92^ ^]^ Copyright 2020, Chen et al. American Association for the Advancement of Science. d) PXRD pattern of the (BA)_2_(MA)_3_Pb_4_I_13_ and (BA)_2_(MA)_4_Pb_5_I_16_ films. e) EL spectra of quasi‐2D perovskite films with different *n*‐values. f) GIWAXS images of the (BA)_2_(MA)_3_Pb_4_I_13_ film fabricated by the hot casting method at RT and 110 °C. g) Illustration of the proposed mechanism of well‐oriented quasi‐2D PeLED via the hot casting method. Reproduced with permission.^[^ ^93^ ^]^ Copyright 2018, Wiley‐VCH.

In addition to utilizing exciton funneling features in quasi‐2D PeLEDs, there have also been reports on phase‐pure quasi‐2D PeLEDs. Although they lack a cascading energy structure, their key advantage lies in the ability to modulate the *n*‐values precisely, enabling the achievement of highly color‐pure PeLEDs. On this front, Chen et al. achieved phase‐pure quasi‐2D PeLEDs by producing single crystals of (BA)_2_Cs_n‐1_Pb_n_Br_3n+1_ (*n *= 1, 2, and 3) and then dry‐transferring exfoliated nanosheets onto a PEDOT: PSS layer (**Figure** **8**a).^[^ ^92^ ^]^ Albeit an average thickness of 34 nm for the transferred nanosheets, there was significant thickness deviation, with some exceeding 100 nm. Single crystal LEDs with *n *= 2 and 3 phases exhibited electroluminescence (EL) spectra with high color purity, with full width at half‐maximum (FWHM) values of 27.5 and 18.1 nm, respectively (Figure 8b). Despite these achievements, the radiance–voltage curve remained unstable, achieving maximum EQE values of 0.7% at 5.6 V for *n *= 2 and 1.1% at 5.4 V for *n *= 3 (Figure 8c). The poor device performance was attributed to non‐uniform coverage, as illustrated in Figure 8a, resulting in low charge injection efficiency.

While phase‐pure LEDs were successfully demonstrated by transferring single crystals, challenges with thickness uniformity and coverage of the single crystals still persisted. A promising alternative could be hot casting, which produces quasi‐2D perovskites with high phase purity and preferred orientation.^[^ ^93^ ^]^ Phase‐pure (BA)_2_(MA)_3_Pb_4_I_13_ (Pb_4_, *n *= 4) and (BA)_2_(MA)_4_Pb_5_I_16_ (Pb_5_, *n *= 5) were produced and confirmed by High‐resolution powder X‐ray diffraction (PXRD) (Figure 8d). This high phase purity enables tuning the EL emission wavelength from 616 to 744 nm depending on the *n*‐value (Figure 8e). The effectiveness of the hot casting method at various temperatures was examined for the quasi‐2D perovskite with *n *= 4. From RT‐casted to 110 °C films, the ring pattern in GIWAXS transformed into distinct Bragg spots (Figure 8f) but reverted to a ring pattern above 130 °C. The Bragg diffraction of (110) and (202) planes suggested vertical growth and alignment of the metal halide slab on the substrate. Excessive temperatures, however, complicated the results due to the low thermal stability of BA and MA. The current density in quasi‐2D PeLED devices fabricated via the hot casting method at 110 °C was significantly increased. Such efficient charge injection was achieved by forming a conductive channel through the vertical orientation of the quasi‐2D perovskites between the electrodes (Figure 8g).

Synthetic control on enhancing phase purity at intermediate *n*‐values continued. Li et al. synthesized single crystal flakes with uniform thicknesses for *n *= 1, 2, 3, and 4 by varying molecular designs and types of polar solvents.^[^ ^94^ ^]^ In addition, Hou et al. employed a confined growth method to successfully grow single crystals of *n* = 1–6, where the precursor solution was placed between two glass substrates with controlled temperature application.^[^ ^79^ ^]^ Despite ongoing challenges in incorporating these methods into LED devices, these studies on phase‐pure film formation hold potential for future LED applications.

## 5 Multiexciton Processes

The unique photophysical properties of quasi‐2D perovskite films, such as high exciton binding energy and cascade energy transfer, make them attractive candidates for LED emitters. However, with increasing current density, quasi‐2D PeLEDs suffer from significant EQE roll‐off, even more severely than their 3D counterparts. In general, multiple factors in the fabrication and operation of LEDs are suspected to cause the EQE roll‐off, such as joule heating,^[^ ^95^ ^]^ imbalanced charge injection,^[^ ^96^, ^97^ ^]^ and ion migration.^[^ ^98^, ^99^ ^]^ In this section, we will focus on the effect of Auger recombination, an intrinsic many‐body photophysical process that most significantly results in the performance difference between quasi‐2D and 3D PeLEDs.^[^ ^100^ ^]^

**Figure 9:**
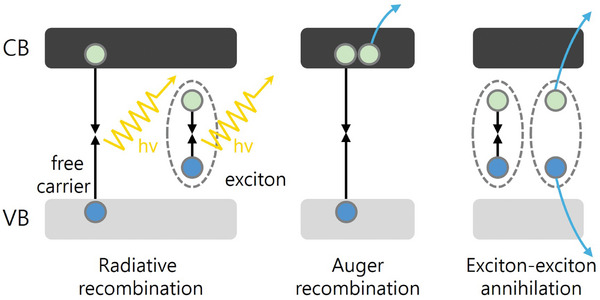
Schematics of radiative recombination, Auger recombination, and exciton–exciton annihilation process.

First, we would like to clarify the terminology. The high exciton binding energies in 2D and quasi‐2D perovskites with low *n*‐values generally dictate the formation of excitons as the main emissive species. In quasi‐2D perovskites with high *n*‐values or unconventional organic spacers, as the degree of dielectric confinement decreases, free carrier and exciton recombination may coexist.^[^ ^37^ ^]^ Within the PeLED field, multibody non‐radiative recombination is commonly summarized under the “Auger recombination” term, which may include the actual Auger recombination which describes the trimolecular process between free carriers as well as exciton–exciton annihilation (EEA) which is a bimolecular effect (**Figure** **9**). Both processes involve the scattering between multiple free carriers or excitons, causing non‐radiative recombination that excites one electron or exciton to a high energy level above the band gap. It should be noted that these two processes are related but fundamentally different; however, they similarly affect the performance of PeLEDs. Beyond the negative impact on device performance, other photophysical processes may require rigorous distinction between Auger recombination and EEA.^[^ ^101^ ^]^ The distinction would typically require kinetic modeling, which is beyond the scope of this review.^[^ ^102^ ^]^ For the sake of convention in the LED community, in this section, we will refer to both effects as Auger recombination.

### 5.1 Influence of Dielectric Confinement on Auger Recombination Rates

We first aim to understand the structural causes leading to the enhanced Auger recombination rates in quasi‐2D perovskites. 3D perovskites already exhibit an increased Auger recombination rate compared with conventional semiconductors due to a coincidental resonance from the conduction band to high‐lying energy states.^[^ ^103^ ^]^ For 2D and quasi‐2D perovskites, the much‐increased dielectric confinement and the resulting large exciton binding energies further promote the nonlinear processes including Auger recombination and EEA.^[^ ^104^, ^105^ ^]^ Power‐dependent PL, time‐resolved PL (TRPL), and TA spectra are the most effective methods for characterizing Auger recombination and EEA rates. Power‐dependent PL spectra reveal how recombination shifts from exciton‐dominated processes at low power to non‐radiative processes like Auger recombination and EEA at higher carrier densities. TRPL and TA both track exciton dynamics but capture different aspects. TRPL focuses on radiative recombination through PL decay, while TA captures both radiative and non‐radiative dynamics by detecting changes in absorption. This makes TA more comprehensive, especially for detecting exciton dissociation, charge carrier separation, and energy transfer, particularly in low‐*n* layered halide perovskites. TA is more suited for ultrafast processes on the picosecond timescale, making it ideal for probing rapid exciton dynamics and nonlinear processes like EEA. Moreover, TA's broader spectral range and greater sensitivity to subtle absorption changes make it particularly well‐suited for investigating complex excitonic and carrier dynamics. In contrast, TRPL excels at tracking slower radiative processes over longer timescales, typically in the nanosecond to microsecond range. While both techniques provide valuable insights, TA is more effective for probing fast exciton and non‐radiative processes, whereas TRPL excels in capturing longer‐term radiative decay behaviors. Although these techniques rely on optical excitation, which differs from electrical excitation in practical PeLED applications, the underlying principles of exciton generation, transport, and recombination remain fundamentally the same. Thus, the insights gained from optical studies are directly relevant to improving device performances.

**Figure 10:**
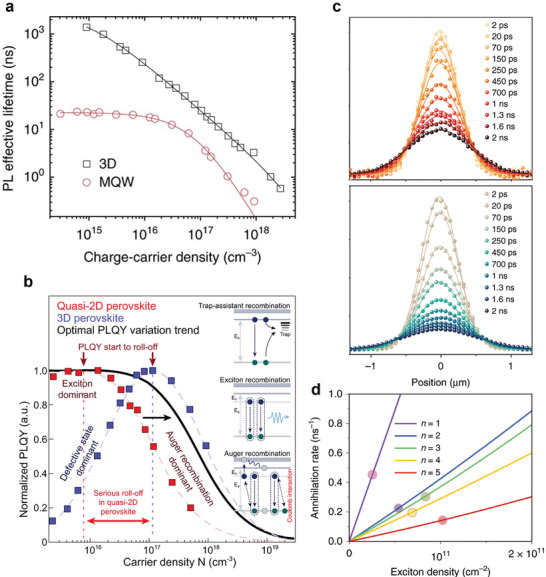
a) PL lifetime and quantum yields in 3D perovskite and quasi‐2D perovskite thin films as a function of carrier density. “3D” represents FAPbI_3_ and “MQW” represents (NMA)_2_FAPb_2_I_7_, where NMA stands for 1‐naphthylmethylammonium. Reproduced under terms of the CC‐BY license.^[^ ^35^ ^]^ Copyright 2017, Xing et al. Springer Nature. b) Experimental PLQYs of quasi‐2D and 3D perovskites as a function of carrier density. Reproduced under terms of the CC‐BY license.^[^ ^111^ ^]^ Copyright 2021, Jiang et al. Springer Nature. c) Population distribution of BA_2_MA_4_Pb_5_I_16_ at initial exciton densities of 1.5 × 10^11 ^cm^−2^ (top) and 2.4 × 10^12 ^cm^−2^ (bottom). d) Total Auger annihilation rate as a function of 2D perovskite thickness, marked in line. The radiative recombination rates are marked in circles for comparison purposes. Reproduced under terms of the CC‐BY license.^[^ ^108^ ^]^ Copyright 2020, Deng et al. Springer Nature.

As the aforementioned comparison between power‐dependent PLQY between quasi‐2D and 3D perovskite thin films pointed to their different behavior at low carrier density, similar trends could be observed in PL lifetime dynamics (**Figure** **10**a). These studies show that quasi‐2D samples also exhibit carrier density‐independent PL lifetime at low carrier densities due to the exciton‐dominated energy landscape, whereas the PL lifetime of 3D perovskites monotonically decreases with increasing carrier density as a result of the increased recombination rate for the bimolecular recombination of free carriers. Notably, quasi‐2D perovskites showed a significant drop in PL lifetime when the carrier density is larger than 10^16^ cm^−3^, as a result of non‐radiative Auger recombination. The density‐dependent PLQYs of quasi‐2D perovskite and 3D perovskite exhibit similar trends, with the quasi‐2D perovskites starting to exhibit suppressed PL efficiency at lower carrier density, reflecting a much faster Auger recombination rate (Figure 10b).

Quasi‐2D perovskites benefit from a much larger compositional space compared with their 3D counterparts. Both the quantum well thickness and the organic spacer can be designed to fine‐tune their photophysical properties.^[^ ^106^ ^]^ Here we will briefly discuss the effects of these two factors on the Auger recombination rates. Density‐dependent TRPL measurements of BA_2_MA*_n_*_–1_Pb*_n_* I_3_ *_n_*_+1_ quasi‐2D thin films are mostly exciton‐dominated, with a small free‐carrier contribution at high average quantum well thickness.^[^ ^107^ ^]^ Studies have shown that the EEA rate correlated strongly with the amount of BA in BA_2_MA*_n_*_–1_Pb*_n_* I_3_ *_n_*_+1_ quasi‐2D thin films, increasing from 0.75 × 10^−10^ to 60 × 10^−10^ cm^3 ^s^−1^ from *n *= 4 to *n *= 1. In their phase‐pure single crystals with *n *= 1–5, Deng et al. further explored the effect of thickness on transport and the many‐body effects by TA microscopy, which mapped the exciton population both temporally and spatially.^[^ ^108^ ^]^ The perovskite single crystals were pumped with a near‐resonance excitation and probed with a scanning beam at the excitonic resonance. Higher carrier density near the center of the pump beam results in relatively faster recombination than at the edge of the pump beam because of EEA and the Auger effect, resulting in an apparent broadening of the TA microscopy signal, whose rate is sensitive to the initial carrier fluence. On the other hand, the diffusion rate of exciton is independent of the initial carrier density. Power‐dependent experiments were conducted to decouple the broadening nonlinear effect from the exciton diffusion (Figure 10c). The diffusion constant increased from 0.06 to 0.34 cm^2 ^s^−1^ from *n *= 1 to *n *= 5, where the Auger and EEA rates decrease over tenfold in such a range. This suggests that high *n* quasi‐2D perovskites are less susceptible to Auger recombination than 2D and low‐n quasi‐2D perovskites (Figure 10d). Li et al. characterized the power‐dependent carrier dynamics in 2D and quasi‐2D Sn‐based perovskites (PEA_2_MA*_n_*_–1_Sn*_n_* I_3_ *_n_*_+1_, *n *= 1–4).^[^ ^109^ ^]^ Surprisingly, despite the lower exciton binding energy compared with Pb‐based perovskites,^[^ ^110^ ^]^ the PL intensity of Sn‐based candidates showed linear relationships with carrier density, and the TRPL lifetime overall did not change, revealing an exciton‐dominated PL recombination. The EEA remains insignificant within the measured pump fluence range, because of pump fluence being likely lower than the carrier density threshold for the multimolecular effects to take place.

### 5.2 Influence of Organic Spacers on Auger Recombination Rates

**Figure 11:**
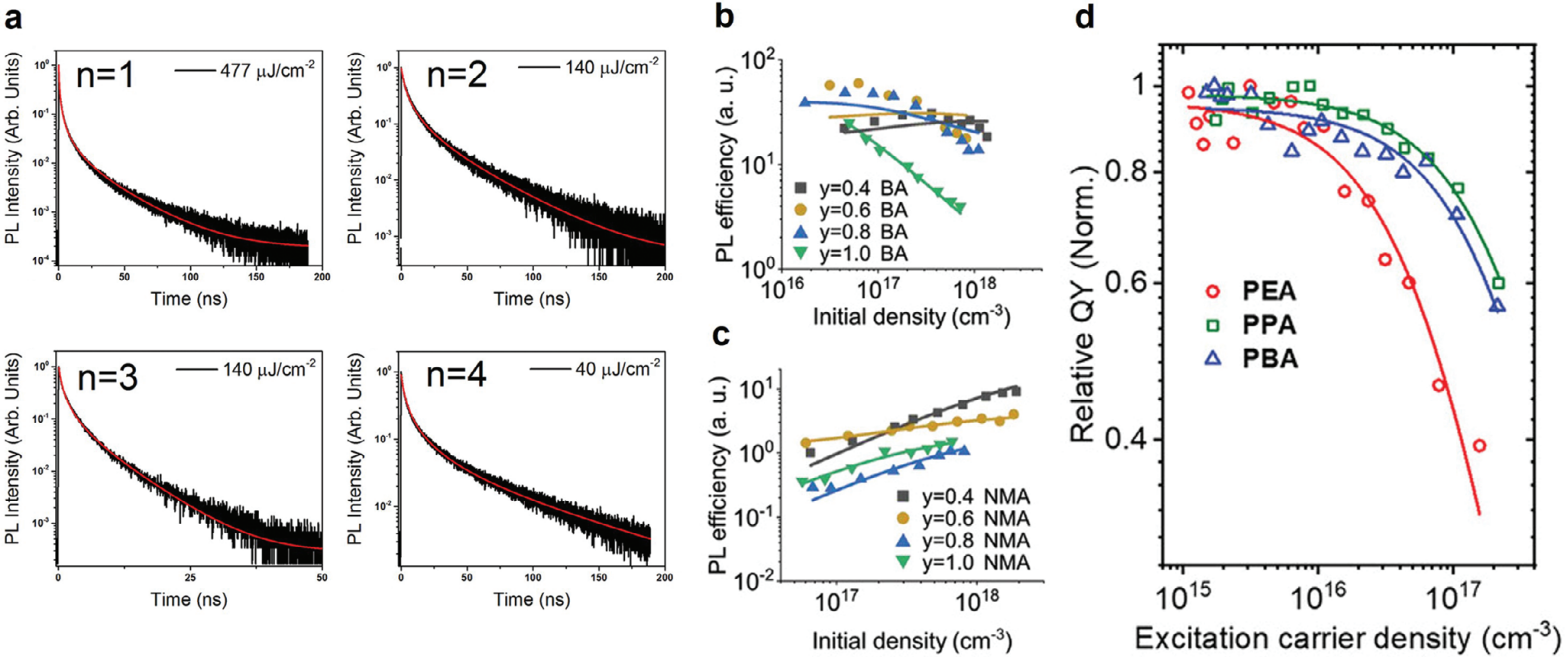
a) PL decay at high excitation power for *n *= 1–4 PEA‐based layered halide perovskite single crystals. Reproduced with permission.^[^ ^37^ ^]^ Copyright 2019, American Chemical Society. Density‐dependent PL efficiency of b) BA‐based and c) NMA‐based 2D and quasi‐2D perovskite thin films. Reproduced under terms of the CC‐BY license.^[^ ^107^ ^]^ Copyright 2021, Kaiser et al. Wiley‐VCH. d) Relative quantum yield for PEA_2_PbBr_4_, PPA_2_PbBr_4_ and PBA_2_PbBr_4_ films. Reproduced with permission.^[^ ^115^ ^]^ Copyright 2024, Wiley‐VCH.

Regarding the organic spacers, in 2019, Delport et al. investigated the exciton dynamics of PEA‐based *n *= 1–4 perovskites in comparison with BA‐based crystals.^[^ ^37^ ^]^ The PEA‐based quasi‐2D structures exhibited mixed exciton and free‐carrier characters in the radiative recombination. The global fitting of density‐dependent TRPL data revealed an increasing radiative recombination rate from *n* = 1 to 4, except for *n* = 3 (**Figure** **11**a), unlike the BA‐based quasi‐2D perovskites. The abnormality in the *n* = 3 sample seemed to originate from the synthetic difficulty of obtaining a low‐defect, phase‐pure sample. Meanwhile, the Auger recombination rates were roughly one order of magnitude lower than that of BA‐based quasi‐2D perovskites, (1.1–4.9 × 10^−4^ cm^2 ^s^−1^ for PEA, 1.3–4.1 × 10^−3^ cm^2 ^s^−1^ for BA),^[^ ^108^ ^]^ with very weak correlation with the quantum well thickness. The weak correlation was partially supported by TA studies on similar PEA‐based perovskite single crystals also showing weakly varying recombination rates in *n *= 2–4 phases albeit rapid bimolecular and trimolecular recombination in 2D perovskites.^[^ ^36^ ^]^ The obvious difference between PEA‐ and BA‐based perovskites could be rationalized by the high dielectric constant in the former, resulting in slightly lower exciton binding energy and less significant Auger recombination.^[^ ^112^, ^113^ ^]^ The impact of the chemical nature of the spacers is more significant in naphthylmethylamine (NMA)‐based organic spacers with larger conjugated system, where their quasi‐2D perovskite emission was dominated by free carriers, and the EEA and Auger recombination are inconsequential compared with defect filling at the measured carrier density (Figure 11b,c).^[^ ^107^, ^114^ ^]^

Another channel of inorganic–organic interactions lies in the influence of spacers on the exciton‐ and electron–phonon coupling in quasi‐2D perovskites. The Auger recombination rates of PEA, phenyl propylammonium (PPA), and phenyl butylammonium (PBA)‐based 2D perovskites were investigated by TA and TRPL spectroscopy.^[^ ^115^ ^]^ PEA‐based films exhibit a rapid decrease in relative PL efficiency with increasing carrier density (Figure 11d). TA dynamics fitting revealed a more than tenfold faster Auger recombination rate for PEA‐based films compared with PPA‐ and PBA‐based films. Given the similar chemical structure, the dielectric environment difference remained insignificant. Instead, the spacer effect was mainly attributed to the stronger exciton–phonon coupling in the PPA and PBA films compared to the PEA film, which reduces the exciton diffusion and thus the probability of the EEA process. Eventually, spacers affect the defect density and stability of perovskite films and inevitably impact the Auger recombination rates.^[^ ^115^, ^116^ ^]^

### 5.3 Mitigating Auger Recombination in Quasi‐2D PeLEDs

**Figure 12:**
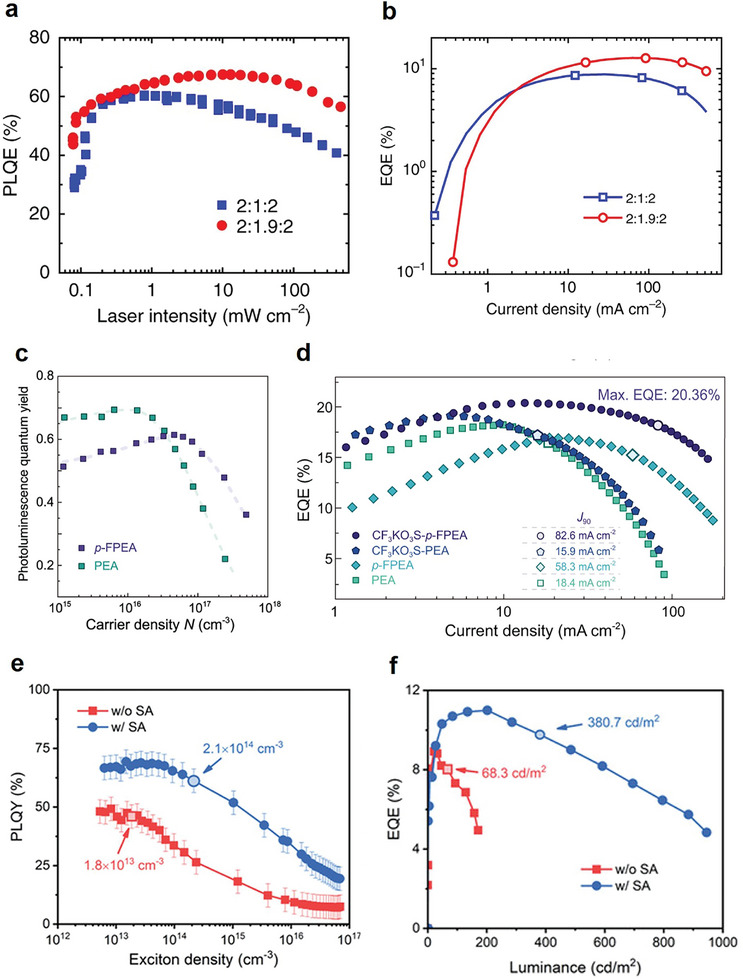
a) Power‐dependent PLQE of quasi‐2D perovskite films with different precursor ratios. b) EQE as a function of current density. The ratio denotes the precursor ratio of NMAI, FAI, and PbI_2_. Reproduced under terms of the CC‐BY license.^[^ ^117^ ^]^ Copyright 2018, Zou et al. Springer Nature. c) Carrier density‐dependent PLQY for PEA‐ and FPEA‐based quasi‐2D films. d) Current density‐dependent EQE for different quais‐2D PeLEDs. Reproduced under terms of the CC‐BY license.^[^ ^111^ ^]^ Copyright 2021, Jiang et al. Springer Nature. e) Power‐dependent PLQY for quasi‐2D perovskite films with and without solvent annealing. f) Luminance‐dependent EQE. Reproduced with permission.^[^ ^119^ ^]^ Copyright 2022, Wiley‐VCH.

The Auger recombination rate is expected to be much more influential in PeLEDs than in perovskite solar cells, given the orders of magnitude higher carrier density in the former. Unfortunately, to date, device‐oriented research on Auger recombination has been largely overshadowed by defect passivation and operational stability. Some strategies for suppressing EQE roll‐off by reducing the intrinsic Auger recombination rate have been explored, through adjusting the composition and spacers of the quasi‐2D emitters as discussed previously. These strategies were pioneered by Zou et al., by adjusting the quantum well mean thickness (and thus exciton binding energy) in NMA‐based quasi‐2D near‐infrared PeLEDs (**Figure** **12**a,b).^[^ ^117^ ^]^ The PeLED devices with optimized quantum well thickness reached a high average EQE of 9.4% at 300 mA cm^−2^, compared with the EQE of 4% of the unoptimized devices. Similarly, the binding energy can be tuned through the spacer polarity using p‐FPEA^[^ ^111^ ^]^ or the spacer lengths in Dion–Jacobson‐phase PeLEDs to effectively suppress Auger recombination at high voltage (Figure 12c,d).^[^ ^118^ ^]^ Arguably, tuning the fabrication conditions and device interfaces might be a more ideal approach to avoid collateral damage to the luminescent or transport properties of the PeLED emitter. For example, it was found that solvent annealing increases the grain size, which in turn suppresses the effective carrier density in quasi‐2D PeLEDs. This process raised the carrier density threshold for significant Auger recombination from 1.8 × 10^13^ to 2.1 × 10^14^ cm^−3^. Together with surface passivation by CsAc, the EQE of PeLED devices showed significantly higher luminance, EQE, and enhanced stability (Figure 12e,f).^[^ ^119^ ^]^

## 6 Triplet Exciton Dynamics in Quasi‐2D Perovskite LEDs

### 6.1 Singlet and Triplet Excitons in Quasi‐2D Perovskites

In organic LEDs (OLEDs), the recombination of uncorrelated electrons and holes spontaneously generates 75% triplet excitons and 25% singlets, according to spin‐statistics. Efficient OLEDs, therefore, necessitate strategic harvesting of triplet excitons.^[^ ^120^, ^121^, ^122^, ^123^, ^124^ ^]^ This is crucial due to the typically non‐radiative nature of triplet excitons (dark excitons) in organic emitters dominated by light elements. However, heavy Pb or Sn atoms in halide perovskites introduce strong spin–orbit coupling, leading to spin mixing and potentially allowing for the full utilization of both spin states.^[^ ^125^, ^126^ ^]^ While 3D perovskites do not need to consider spin–orbit coupling since their light emission mechanisms involve free carrier recombination,^[^ ^127^ ^]^ the scenario changes with layered halide perovskites, which are excitonic recombination‐dominant and contain a high proportion of organic compounds.^[^ ^13^, ^23^ ^]^ This necessitates addressing the exciton dynamics of different spin states in the context of organic spacers. This section will elaborate on these dynamics and their implications for PeLED efficiency.

**Figure 13:**
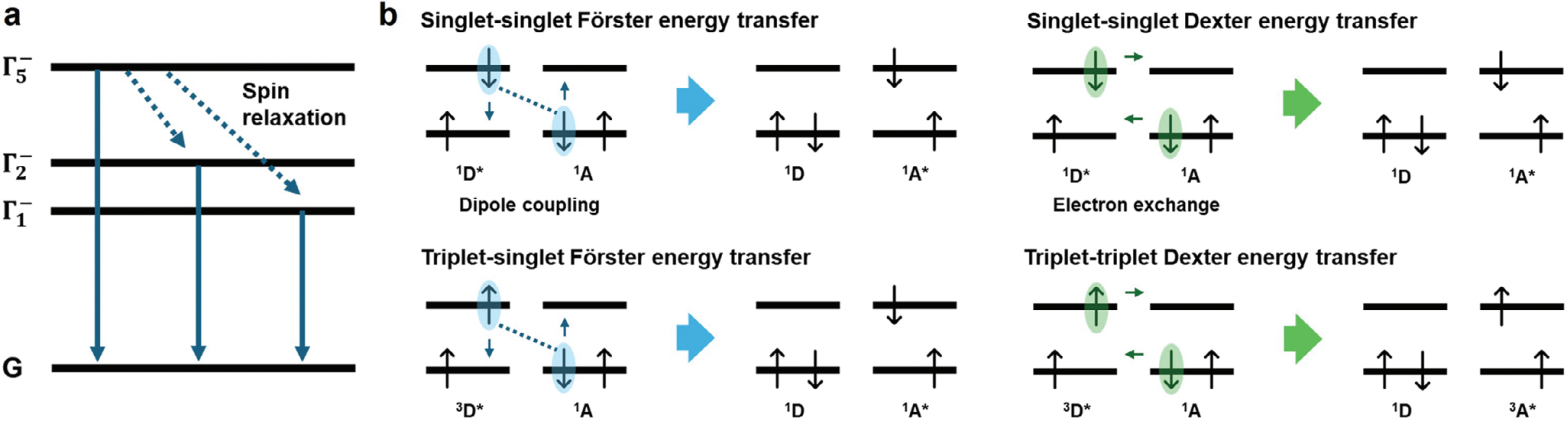
Schematics of a) (C_4_H_9_NH_3_)_2_PbBr_4_ 2D perovskite exciton binding energy levels and relaxation processes (Reproduced with permission.^[^ ^128^ ^]^ Copyright 2006, American Physical Society), and b) FRET and DET mechanisms with different spin states.

The spin of an exciton inherits the spins of bound electron and hole. An exciton can exist as a spin‐antiparallel singlet state, which does not alter the spin configuration in the ground state, resulting in a total spin angular momentum *S* = 0 (calculated by adding the individual spin angular momentum with spin up as *s* = 1/2 and spin down as *s* = −1/2). Alternatively, an additional spin‐flip can create a spin‐parallel electron–hole pair, resulting in a triplet state with *S* = 1. The terms “singlet” and “triplet” originate from the spin multiplicity 2*S *+ 1. Thus, triplet states contain three sublevels, and their degeneracy can be lifted by factors such as internal or external magnetic fields. In (C_4_H_9_NH_3_)_2_PbBr_4_ 2D perovskites, for instance, the inorganic layer [PbBr_4_]^2−^ exhibits a *Γ*_5_ singlet state at 3.014 eV and two triplet states, *Γ*_1_ and *Γ*_2_, at 2.989 and 2.991 eV, respectively.^[^ ^22^, ^128^, ^129^ ^]^ The small single–triplet gaps originate from effective dielectric screening and hence low electron exchange energy. Although *Γ*_1_ and *Γ*_2_ excitons are dark excitons and typically dipole forbidden, their emissions can be observed because of crystal distortion, higher‐order transition moment processes, and phonon‐assisted transitions that break the selection rule.^[^ ^128^ ^]^ In terms of emission intensity, *Γ*_1_ and *Γ*_2_ triplet excitons show stronger emission than the *Γ*_5_ singlet emission because of fast spin relaxation from *Γ*_5_ to *Γ*_1_ and *Γ*_2_, leading to the increase in populations of *Γ*_1_ and *Γ*_2_ excitons (**Figure** **13**a).^[^ ^128^ ^]^ Thus, managing triplet excitons is essential for designing efficient quasi‐2D PeLEDs. To achieve this, we need to first consider the energy transfer processes involving different spin states (Figure 13b).

### 6.2 Energy Transfer Mechanisms

The Förster resonance energy transfer (FRET) is a long‐range, non‐radiative energy transfer mechanism that an excited donor transfers energy to an acceptor in its ground state through a non‐radiative dipole coupling.^[^ ^130^ ^]^ The efficiency of FRET is inversely proportional to the distance between the donor and acceptor, and it is typically most effective within 10 nm.^[^ ^131^ ^]^ The efficiency of FRET is given by Equation (1):

(2) $$E=\frac{1}{1+{\left(\frac{R_{0}}{r}\right)}^{n}}\;$$

where *R_0_* is the distance between donor and acceptor, *r* is the Förster distance at which the transfer efficiency is 50%, and *n* is the power that depends on the dimensional nature of the dipoles involved (e.g., *n* = 6 for point dipoles). FRET efficiency generally depends on the dipole resonance between donor and acceptor and can be affected by the spectral overlap between donor emission and acceptor absorption and their dipole orientation alignment besides the aforementioned separation distance. Considerable donor PLQY and low refractive index of the medium are two other general considerations.

On the other hand, Dexter energy transfer (DET) is a short‐range, non‐radiative energy transfer process that occurs via electron exchange between the donor and acceptor groups.^[^ ^132^ ^]^ It is effective only over very short distances, typically less than 1–2 nm. The efficiency of DET decreases exponentially with distance, as shown in the following equation:

(3) $$k_{D}=k_{0}\;e^{-\frac{2R}{L}}$$

where *k_D_* is the DET rate, *k_0_* is a prefactor, and *R* is the distance between donor and acceptor. The equation points to the need for a significant overlap of the electron wavefunctions between the donor and acceptor candidates as expressed by *L*, the characteristic decay length of the wavefunction overlap.

DET involves the exchange of electrons, making it capable of transferring both singlet and triplet excitons by facilitating necessary spin flip. However, most research on FRET emphasizes this process is primarily effective for singlet excitons due to the dipole–dipole coupling inherent in the allowed transitions between singlet states.^[^ ^133^, ^134^ ^]^ Nonetheless, in cases where spin–orbit coupling is strong, the oscillating strength of the triplet cannot be neglected, which allows triplet states to participate in the FRET mechanism.^[^ ^135^, ^136^, ^137^ ^]^

### 6.3 Triplet Exciton Management in Quasi‐2D Perovskite LEDs

**Figure 14:**
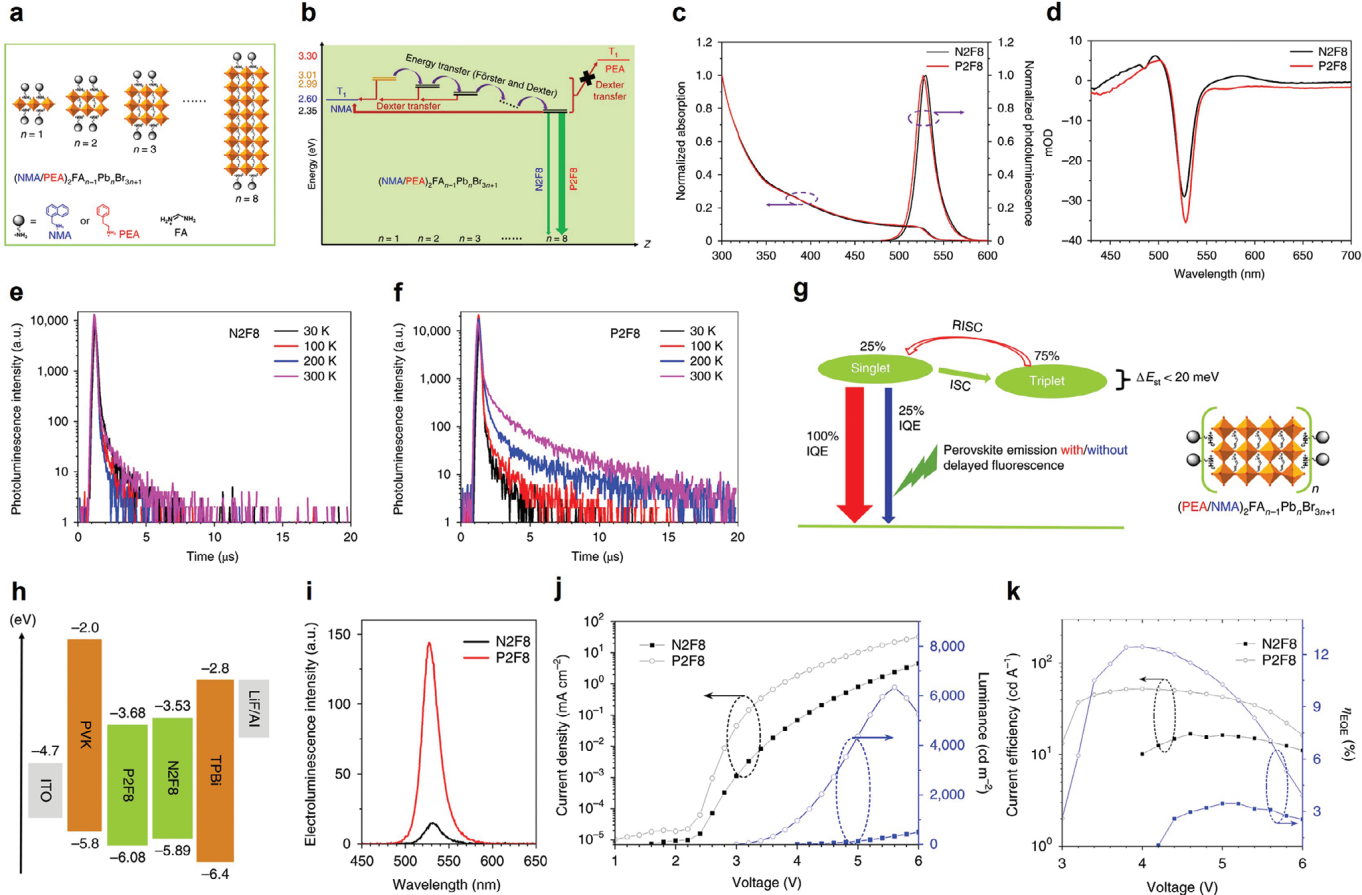
a) Chemical and unit cell structures of the 2D and quasi‐2D perovskites with PEA and NMA. b) Proposed energy transfer mechanism. c) Steady‐state absorption, PL spectra, and d) TA spectra of N2F8 (NMA) and P2F8 (PEA) at room temperature. Temperature‐dependent PL spectra of e) N2F8 and f) P2F8. g) Delayed fluorescence mechanism. h) Device structure of the PeLEDs. i) EL spectra of the PeLEDs at 5 V. (j) *J*–*V*–*L* curves, and k) EQE and current efficiency characteristics of the PeLEDs. Reproduced with permission.^[^ ^138^ ^]^ Copyright 2020, Springer Nature.

Based on such energy transfer characteristics of different spin states, Adachi's group demonstrated the enhancement of EQE in quasi‐2D green PeLEDs by harvesting triplet excitons through organic spacer engineering.^[^ ^138^ ^]^ PEA and NMA were combined with FAPbBr_3_ precursor solution to produce two similar quasi‐2D perovskites, (P2F8 and N2F8, respectively) (**Figure** **14**a). As illustrated in Figure 14b, the lowest excited triplet state (*T*_1_) energy of NMA is merely 2.6, 0.4 eV lower than the inorganic triplet exciton energy levels *Γ*_1_ and *Γ*_2_ of 2D perovskite and is also lower or comparable to the triplet states’ energy in quasi‐2D perovskites. Consequently, efficient triplet–triplet DET should occur from inorganic lattices to the *T*_1_ of NMA in quasi‐2D structures due to the proximity of the chemically linked components.^[^ ^22^ ^]^ This energy transfer process competes with the regular cascade energy transfer within MQW structures. In contrast, such an energy quenching does not happen in PEA‐based quasi‐2D perovskites due to a higher *T*_1_ of 3.3 eV in PEA, allowing efficient energy cascade and enabling the full utilization of singlets and triplets. The two films exhibited similar steady‐state absorption and PL spectra of typical quasi‐2D perovskites (Figure 14c). In TA spectra, unlike the P2F8 film, the N2F8 film displayed a positive peak near 580 nm in addition to sharp bleach peaks ≈526 nm (Figure 14d). This additional peak is attributed to triplet–triplet absorption resulting from intermoiety charge transfer absorption of the intramolecular triplet excimer of NMA,^[^ ^139^ ^]^ indicating Dexter triplet energy transfer from N2F8 to the NMA cation. Furthermore, temperature‐dependent TRPL spectra analysis revealed that as the temperature increased to 300 K, the P2F8 exhibited slower PL decay behavior compared to the N2F8 film, which maintained a constant PL decay behavior under varying temperatures (Figure 14e,f). This phenomenon was ascribed to emission arising from the thermally activated reverse intersystem crossing of triplet excitons (Figure 14g).^[^ ^120^ ^]^

With this strategy, they constructed devices with the structure depicted in Figure 14h. The energy band diagram for these devices shows that N2F8 and P2F8 possess similar valence and conduction band levels, indicating a comparable interfacial energetic alignment for hole and electron injection from the Poly (9‐vinylcarbazole) (PVK) and 2,2′,2″‐(1,3,5‐Benzinetriyl)‐tris(1‐phenyl‐1‐H‐benzimidazole) (TPBi) layers, respectively. The N2F8‐ and P2F8‐based devices emit green light with peak wavelengths of 531 and 527 nm, respectively, and share the same FWHM value of 21 nm (Figure 14i). The representative *J*–*V*–*L* characteristics of the PeLEDs at room temperature are exhibited in Figure 14j. The N2F8‐based PeLEDs achieved a maximum luminance of 500 cd m^−2^ at 6 V, whereas the P2F8‐based PeLEDs exhibited a higher brightness of 5200 cd m^−2^ at the same voltage. Additionally, the maximum EQE and current efficiency of the P2F8‐based PeLEDs (12.4% and 52.1 cd A^−1^) were more than three times those of the N2F8‐based devices (3.4% and 16.3 cd A^−1^), as shown in Figure 14j,k. These findings confirm that the varying contributions from singlet and triplet excitons result in different device performances.

**Figure 15:**
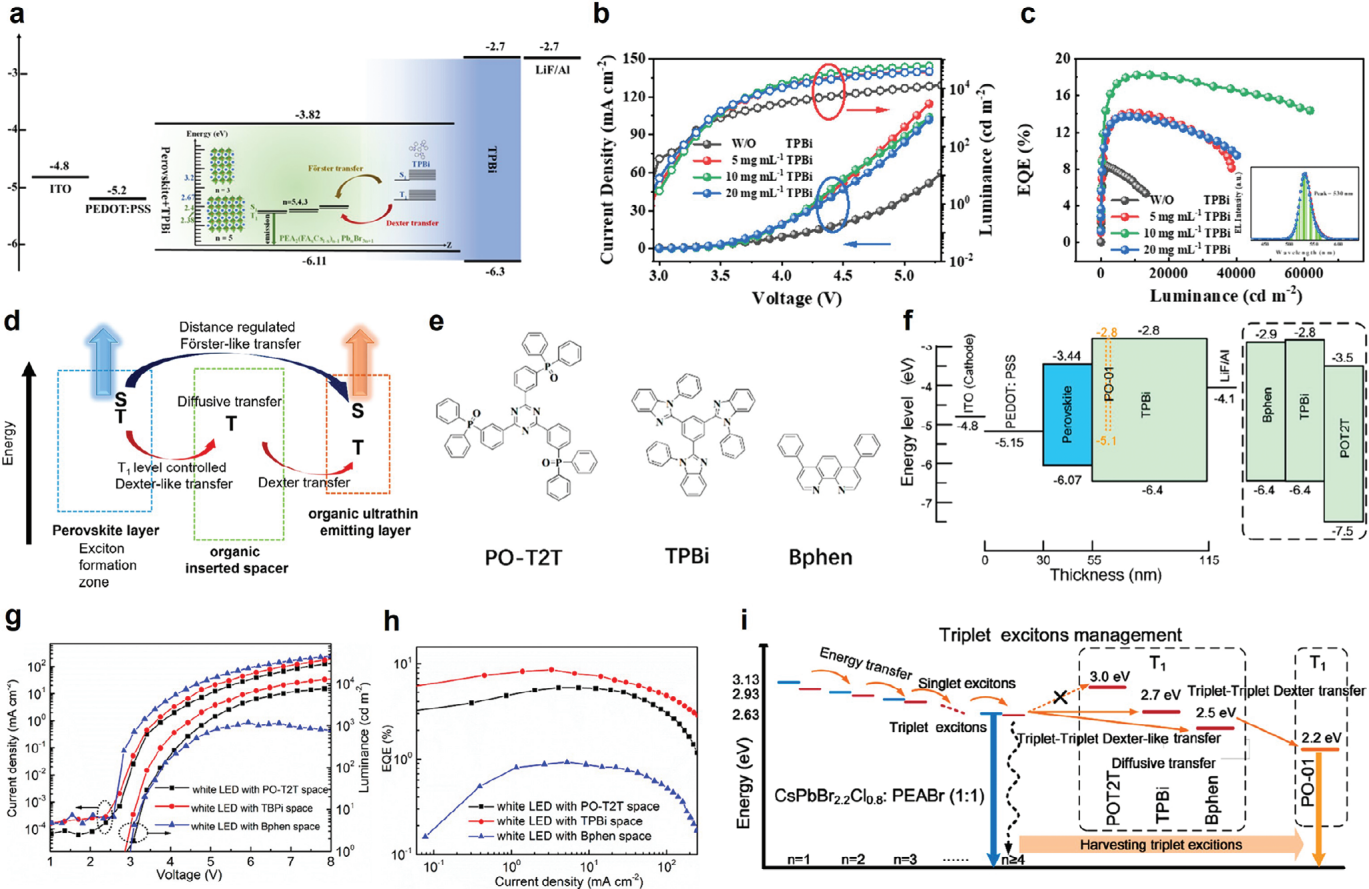
a) Energy level diagram of the PeLEDs and energy transfer mechanism inside the perovskite layer. b) *J*–*V*–*L* curves and c) EQE‐luminance curves of the PeLEDs. Reproduced with permission.^[^ ^140^ ^]^ Copyright 2024, American Chemical Society. d) Proposed excitons management mechanisms in the white LEDs. e) Chemical structures of the electron transport layers (ETLs) with varying triplet energy levels. f) Energy levels of the designed white LEDs. g) *J*–*V*–*L* curves and h) EQE characteristics of the white LEDs with different ETLs. i) Proposed triplet exciton management mechanism of the white LEDs. Reproduced with permission.^[^ ^142^ ^]^ Copyright 2022, American Chemical Society.

Wei et al. recently reported efficient green quasi‐2D PeLEDs using a similar approach.^[^ ^140^ ^]^ They incorporated PEABr as an organic spacer for the quasi‐2D phase and TPBi as the host material in the perovskite emissive layer. As shown in **Figure** **15**a, the *S*_1_ and *T*_1_ energy levels of TPBi are 3.2 and 2.67 eV, respectively, which are both higher than those of the perovskite layer.^[^ ^138^, ^141^ ^]^ Particularly, the higher‐lying *T*_1_ of TPBi effectively confines excitons and prevents exciton quenching. This allows effective energy transfer processes to occur between TPBi and quasi‐2D perovskites and the subsequent energy cascade. To validate this effect on PeLEDs, they fabricated devices with the structure illustrated in Figure 15a. As shown in Figure 15b,c, they achieved high‐efficiency green PeLEDs with an EQE_max_ of 18.26% and an *L*_max_ of 61 704 cd m^−2^, marking a 118.8% increase in the maximum EQE compared to the undoped device.

Another interesting approach in harvesting triplet excitons for white LEDs was reported by Wu's group.^[^ ^142^ ^]^ They utilized a reverse energy transfer from the quasi‐2D perovskite to the organic emitter by adjusting the triplet energy level and thickness of the *n*‐type spacer in the device. As shown in Figure 15d, the inserted spacer plays a significant role in blocking holes and avoiding direct electron–hole recombination at the ultrathin organic layer that leads to concentration quenching. Given that both energy transfer efficiency depends on the distance between donor and acceptor molecules, the energy transfer process could be regulated by altering the thickness of the spacer. For the perovskite layer, a mixture of 2D and 3D perovskites was used with CsPbBr_2.2_Cl_0.8_:PEABr (1:1) compositions. Three different types of materials, (1,3,5‐triazine‐2,4,6‐triyl)tris‐(benzene‐3,1‐diyl))tris(diphenylphosphine oxide) (PO‐T2T), TPBi, and bathophenanthroline (Bphen) were compared as spacers and ETLs with decreasing T_1_ levels of 3.0, 2.7, and 2.5 eV (Figure 15e). A commercial orange bis(4‐phenylth‐ieno[3,2‐c]pyridine) (acetylacetonate)‐iridium(III) (PO‐01) phosphorescent emitter with *T*_1_ level of 2.2 eV, which can harvest both singlet and triplet excitons, was used to prepare an ultrathin emitting nanolayer and examine triplet behavior in perovskite. The designed white LEDs were fabricated with the configuration of ITO/PEDOT: PSS (≈30 nm)/perovskite (≈25 nm)/inserted spacer (*x* nm)/PO‐01 (<1 nm)/ETL (6 − *x* nm)/LiF (1 nm)/Al (100 nm) as described in Figure 15f. The optimized device with 7.5 nm‐thick TPBi spacer and 0.5 nm‐thick PO‐01 ultrathin layer exhibited a maximum forward‐viewing EQE of 8.6% at a current density of 3.3 mA cm^−2^ (Figure 15g,h). The detailed triplet exciton management mechanism is shown in Figure 15i. Both TPBi and Bphen‐based devices showed EQE enhancement due to a well‐designed Dexter‐like triplet energy transfer channel instead of the Forster‐like energy transfer (harvesting triplet excitons in the sky‐blue perovskite). In contrast, the device with a PO‐T2T spacer with high T_1_ energy exhibited no EQE enhancement due to the blocking of the Dexter‐like triplet energy transfer process at the perovskite/PO‐T2T interface.

## 7 Conclusions and Perspectives

In this review, we have explored the critical aspects of exciton dynamics in layered halide PeLEDs, discussing the fundamental structural and photophysical properties of layered halide perovskites, with an emphasis on the unique excitonic behaviors that arise from dimensional reduction and enhanced dielectric confinement. Understanding these properties is crucial for optimizing PeLED performance, as they directly influence exciton binding energy, recombination rates, and the efficiency of radiative and non‐radiative processes. To achieve high‐performance layered halide PeLEDs from an exciton dynamics perspective, the following key factors must be addressed.

- Phase Engineering: Achieving phase purity and mitigating phase separation are essential for high‐performance quasi‐2D PeLEDs. One promising direction is the development of crystallization techniques that limit the formation of low‐*n* 2D phases, which tend to dominate and cause non‐uniform emission. Advanced methods such as hot casting, slow crystallization, and the use of sterically hindered organic spacers have shown great potential in controlling the distribution of *n*‐phases and improving device performance. Furthermore, the molecular design of organic spacers has emerged as an effective strategy to suppress ion migration, thereby stabilizing the phase distribution and enhancing energy funneling efficiency. The exploration of phase‐pure quasi‐2D PeLEDs also holds significant promise, with recent studies demonstrating high color purity and tunable emission through precise modulation of the *n*‐values. However, issues related to thickness uniformity and charge injection efficiency in these devices remain, and further optimization is required. Another key area of future research will be the development of novel additives and molecular spacers that not only enhance phase control but also improve exciton management, leading to more efficient energy transfer and higher EQEs. Continued efforts to refine the crystal orientation and suppress defect formation through careful solvent engineering and post‐treatment techniques will be crucial to pushing the limits of quasi‐2D PeLED performance.
- Auger Recombination and EEA Mitigation: Reducing Auger recombination and exciton–exciton annihilation is vital for improving the performance and stability of quasi‐2D PeLEDs. The degree of dielectric confinement and quantum well thickness in quasi‐2D perovskites has a strong influence on Auger recombination rates. By carefully controlling these parameters, particularly through fine‐tuning the *n*‐phase distribution, non‐radiative losses can be minimized, optimizing the emission characteristics of quasi‐2D PeLEDs. Furthermore, increasing the quantum well thickness in phase‐pure single crystals has shown promise in reducing Auger recombination rates, as demonstrated by recent studies using TA microscopy. Additionally, the choice of organic spacers plays a crucial role in modulating exciton dynamics and recombination processes. Spacers like PEA and NMA have shown potential in reducing Auger recombination rates due to their lower exciton binding energy and dielectric properties. Further research into novel organic spacers that can minimize exciton–phonon coupling and enhance charge carrier mobility will be essential for improving the performance of quasi‐2D perovskites. Beyond material design, device‐level strategies, such as solvent annealing and interface engineering, offer additional pathways to mitigate Auger recombination. By increasing the grain size and improving surface passivation, the effective carrier density can be reduced, thereby delaying the onset of Auger recombination at high current densities. Additionally, exploring Sn‐based quasi‐2D perovskites, which exhibit lower exciton binding energies and exciton‐dominated recombination, could offer promising solutions to Auger recombination challenges.
- Triplet Exciton Management: While less critical than in OLEDs, effective harvesting of triplet excitons through energy transfer mechanisms remains important for improving quasi‐2D PeLED efficiency. Strong spin–orbit coupling in quasi‐2D perovskites can enable efficient radiative recombination from triplet states. Organic spacer engineering, such as tuning the energy levels of spacers like PEA and NMA, has shown potential in optimizing this process, as demonstrated by increased EQE through effective triplet–triplet energy transfer. Organic spacers such as TPBi have been shown to effectively confine excitons, preventing quenching and promoting efficient energy transfer between the perovskite layer and the spacer. Further exploration of spacer materials with optimized triplet energy levels can lead to even greater gains in device performance.

In short, this article provides a comprehensive review from the fundamental properties of excitons in layered halide perovskites to strategies for improving LED device efficiencies and addressing efficiency roll‐off issues. We hope that this work inspires next‐generation researchers to develop novel materials, new device operation mechanisms, and advanced processing techniques toward even higher‐performance PeLEDs.

## Conflict of Interest

The authors declare no conflict of interest.
